# Use of Antidepressant for the Treatment of Depression in CAMHS Outpatient

**DOI:** 10.1192/bjo.2025.10652

**Published:** 2025-06-20

**Authors:** Chidimma Orji, Linda Robinson

**Affiliations:** Lancashire and South Cumbria NHS Foundation Trust, Preston, United Kingdom

## Abstract

**Aims:** According to NICE guidelines children and young people with depression should be treated on an outpatient basis. Antidepressants should not be offered routinely to a child or young person with moderate to severe depression except in combination with a concurrent psychological therapy.

When an antidepressant is prescribed to a child or young person with a depressive disorder, it should be fluoxetine as this is the only antidepressant for which evidence shows that benefits outweigh the risks. There should be monitoring and review of mental state in the course of treatment.

The aim of the audit was to evaluate the use of antidepressants for the treatment of depression in children and young people and the monitoring in place for the duration of treatment at West Lancashire CAMHS outpatient clinic in keeping with NICE guidelines.

**Methods:** Cohort: Outpatients (young persons) at CAMHS outpatient clinic (Westgate House) prescribed antidepressants for the treatment of depression.

Sample size: 15–20 patients (randomly selected).

Data collection: Retrospective data collection looking through patient’s record (RIO) for documented diagnosis of depression, medication initiation process, choice of medication, psychological therapy offered and evidence of monitoring – side effects, review of mental state etc.

**Results:** All the young persons had a documented diagnosis of depressive disorder on their records following psychiatrist review. Medication and side effect profile were discussed with the young person and family prior to initiation.

85% of the young persons were prescribed fluoxetine as the first-line medication whilst sertraline was given to the remaining.

The range of offered psychological interventions together with pharmacological treatment include: distraction techniques, use of bedside box, psychoeducation, coping strategies, art/play therapy, behavioural activation, problem solving group, DBT, CBT, IPT and family therapy.

Monitoring was done by the prescriber and case managers weekly (60%), two weekly (25%) and monthly (10%).

**Conclusion:** The range of psychological interventions offered to the young persons were compared with NICE guidelines. Though DBT is not recommended by the guidelines, it was used as a psychological therapy in a background of trauma and emotional dysregulation.

Sertraline was used as first line in one of the young persons with co-morbid PTSD.

Recommendations made to improve the monitoring using the side effect checklist – before prescribing as stated in NICE guidelines and also to liaise with the case managers in the monitoring process after treatment initiation.

